# Tin-Naphthalene Sulfonic Acid Complexes as Photostabilizers for Poly(vinyl chloride)

**DOI:** 10.3390/molecules26123629

**Published:** 2021-06-14

**Authors:** Hadeer Jasem, Angham G. Hadi, Gamal A. El-Hiti, Mohammed A. Baashen, Hassan Hashim, Ahmed A. Ahmed, Dina S. Ahmed, Emad Yousif

**Affiliations:** 1 Department of Chemistry, College of Science, University of Babylon, Babylon 51002, Iraq; hadeer.abd_alhussain@student.uobabylon.edu.iq (H.J.); sci.angam.ganem@uobabylon.edu.iq (A.G.H.); 2 Cornea Research Chair, Department of Optometry, College of Applied Medical Sciences, King Saud University, P.O. Box 10219, Riyadh 11433, Saudi Arabia; 3 Department of Chemistry, College of Science and Humanities, Shaqra University, Dawadmi 11911, Saudi Arabia; mbaashen@su.edu.sa; 4 Department of Physics, College of Science, Al-Nahrain University, Baghdad 64021, Iraq; hassan.hashim@ced.nahrainuniv.edu.iq; 5 Polymer Research Unit, College of Science, Al-Mustansiriyah University, Baghdad 10052, Iraq; dr_ahmedabd@uomustansiriyah.edu.iq; 6 Department of Medical Instrumentation Engineering, Al-Mansour University College, Baghdad 10067, Iraq; dina.saadi@muc.edu.iq; 7 Department of Chemistry, College of Science, Al-Nahrain University, Baghdad 64021, Iraq; emad.yousif@ced.nahrainuniv.edu.iq

**Keywords:** poly(vinyl chloride), tin-naphthalene sulfonic acid complexes, photostabilizers, weight loss, ultraviolet irradiation, thin films, surface morphology, photodegradation

## Abstract

Poly(vinyl chloride) degrades when exposed to ultraviolet light for long durations; therefore, the photostability of polymeric materials should be enhanced through the application of additives. New organotin complexes containing 4-aminonaphthalene-1-sulfonic acid were synthesized and their role as poly(vinyl chloride) photostabilizers were evaluated. The reaction of 4-amino-3-hydroxynaphthalene-1-sulfonic acid and appropriate di- or trisubstituted tin chloride (triphenyltin chloride, tributyltin chloride, dibutyltin dichloride, and dimethyltin dichloride) in methanol under reflux gave the corresponding tin-naphthalene complexes with yields of 75%–95%. Elemental analyses and spectroscopic techniques including infrared and nuclear magnetic resonance (proton and tin) were used to confirm their structures. The tin complexes were added to poly(vinyl chloride) to produce thin films that irradiated with ultraviolet light. Various parameters were assessed, such as the weight loss, formation of specific functional groups, changes in the surface due to photoirradiation, and rate constant of photodegradation, to test the role played by the organotin complexes to reduce photodegradation in polymeric films. The results proved that organotin complexes acted as photostabilizers in these circumstances. The weight loss, formation of fragments containing specific functional groups, and undesirable changes in the surface of polymeric films were limited in the presence of organotin complexes. Organotin complexes containing three phenyl groups showed the most desirable stabilization effect. These act as efficient primary and secondary photostabilizers, and as decomposers for peroxides. In addition, such an additive inhibits the dehydrochlorination process, which is the main cause of poly(vinyl chloride) photodegradation.

## 1. Introduction

Plastics are large molecular mass polymers that have very useful properties that enable their adaptation to various applications. They are strong, durable, stable, can be molded at a high temperature, and can resist fire, weather, and chemicals [1,2]. Plastics can be used in sports and medical equipment, construction, automotive manufacturing, electronics, toys, packaging, bottles, containers, and plastic bags. Poly(vinyl chloride) (PVC) is a common thermoplastic polymer that is commonly produced on a commercial scale [3]. In terms of production and consumption, PVC is second to polyolefins due to its unique properties and low production cost [4]. PVC is produced in various forms mainly as a flexible (plasticized with a low degree of crystallinity) or rigid (non-plasticized with a high degree of crystallinity) material [5]. It has unique properties; however, it suffers from weathering due to long term exposure to ultraviolet (UV) light, humidity, and high temperatures (above 100 °C) [6,7]. It is considered that UV light is the main cause of PVC photodegradation. The mechanism by which PVC photodegradation takes place is not well documented and various speculations have been made [8,9]. The photodegradation of PVC leads to the formation of small polyene fragments, a decrease in molecular mass, a reduction in durability and strength, cracks, discoloration, and loss of mechanical properties [10,11,12]. Various additives have been added to PVC to increase its photostability and life time. These PVC additives mainly act as UV absorbers, radical decomposers, and energy quenchers [13,14]. Therefore, suitable photostabilizers should be mixed with PVC to prevent its decomposition and photooxidation. Such additives can preserve the mechanical and physical properties of PVC for a long time. These additives should be safe to use, not alter the color, blend well with the polymer, and be non-toxic and non-volatile. Recent research has concentrated on the development and use of new inexpensive PVC photostabilizers that contain aromatic moieties at a low concentration [15,16]. The most common PVC additives include Schiff bases containing aromatic moieties such as thiophene [17], 1,2,4-triazole [18], thiadiazole [19,20], and 4*H*-1,2,4-triazole-3-thiol [21]. They also include pigments [14], titanium dioxide [22,23], zinc oxide [24], polyphosphates [25], and polybenzimidazoles [26].

Organotin compounds are chemically and physically stable, and have been used in various pharmaceutical and medicinal applications [27]. For example, they act as antivirals against cancer [28], inflammatory agents [29], antimicrobials [30], and anti-antitubercular agents [31]. In addition, they can be used as preservatives for wood, biocides, and catalysts [32,33]. Organotin compound performance was found to be dependent on the substituents attached to the tin in terms of their types (aliphatic, aromatic, or heterocyclic) and number [34]. Recently, we investigated the role played by tin complexes of mefenamic acid [35], carvedilol [36], and sodium fusidate [37] as PVC photostabilizers. In the current research, we report the design, synthesis, and use of new tin complexes of 4-amino-3-hydroxynaphthalene-1-sulfonic acid as additives for PVC to reduce its photooxidation and photodegradation. These complexes are expected to work as efficient PVC photostabilizers compared with those reported because they are highly aromatic (e.g., naphthalene ring) and contain heteroatoms such as nitrogen, oxygen, and sulfur.

## 2. Results and Discussion

### 2.1. Synthesis of Tin-Naphthalene Sulfonic Acid Complexes ***1***–***4***

**Scheme 1:**
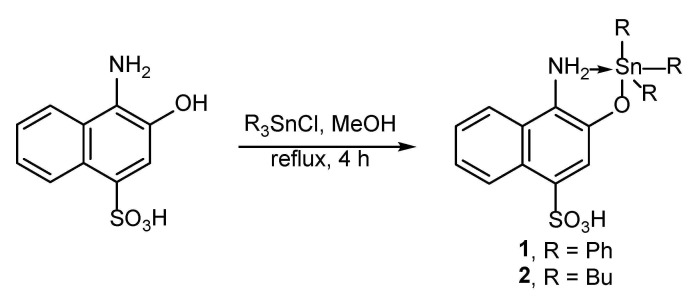
Synthesis of tri-substituted tin-naphthalene sulfonic acid complexes **1** and **2**.

**Scheme 2:**
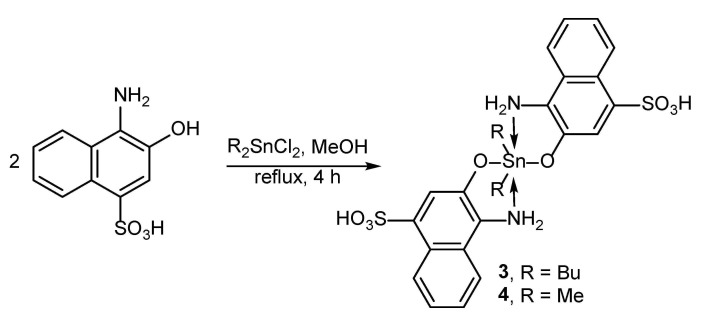
Synthesis of di-substituted tin-naphthalene sulfonic acid complexes **3** and **4**.

Four substituted tin-naphthalene sulfonic acid complexes were synthesized in high yields using a simple procedure. Reaction of a 1:1 mixture of 4-amino-3-hydroxynaphthalene-1-sulfonic acid (ligand) and triphenyl- and tributyltin chlorides in refluxing methanol (MeOH) for 4 h resulted in the corresponding trisubstituted tin complexes **1** and **2** with 65% and 70% yields, respectively (Scheme 1). Similarly, the reaction of the ligand (two mole equivalents) and dibutyl- and dimethyltin dichlorides resulted in disubstituted tin complexes **3** and **4** with 95% and 89% yields, respectively (Scheme 2). The yields (%), color, and melting points (MPs) of tin-naphthalene sulfonic acid **1**–**4** are reported in Table 1. The purity of materials was established by the determination of the content (%) of carbon, hydrogen, sulfur, and nitrogen (Table 1).

### 2.2. IR Spectroscopy of Tin-Naphthalene Sulfonic Acid Complexes ***1***–***4***

The infrared (IR) spectra of **1**–**4** have distinguished bands that appeared within the regions of 3240–3314 cm^−1^, 1597–1611 cm^−1^, and 1526–15301 cm^−1^, which are related to the vibrations of the NH_2_, C–N, and C–O groups, respectively (Table 2). The high shift seen in the wave length of the vibrations of these groups compared with those for the ligand proved that the complexation between the ligand and tin had taken place [38]. In addition, the disappearance of the stretching vibration of the O–H group in the tin complexes, which appeared at 3442 cm^−1^ for the ligand, provides further evidence of the complexation. New bands corresponding to the stretching vibrations of the Sn–C (515–518 cm^−1^) and Sn–O (455–518 cm^−1^) were seen in the IR spectra of **1**–**4**. The assignments of these peaks were consistent with those reported for related compounds [39,40]. No shift was seen in the symmetric and asymmetric vibrations of the SO_2_ group in complexes **1**–**4** compared with those for the ligand.

### 2.3. NMR Spectroscopy of Tin-Naphthalene Sulfonic Acid Complexes ***1***–***4***

The ^1^ H and ^119^ Sn NMR spectral data of tin-naphthalene sulfonic acid complexes **1**–**4** confirmed their structures (Table 3). The ^1^ H NMR spectra showed the presence of two exchangeable broad singlets that appeared within the regions of 11.01–11.06 ppm and 9.78–9.84 ppm corresponding to the SO_3_H and NH_2_ protons, respectively. They also showed the presence of protons corresponding to naphthyl and substituent groups. The ^119^ Sn NMR spectra showed the presence of a singlet that appeared at the −167.0 ppm to the −274.1 ppm region. The chemical shifts for the tin atom indicated that the coordination number for the complexes **1** and **2** is five, whereas complexes **3** and **4** have a coordination number of six [41,42,43].

### 2.4. Weight Loss of PVC

**Figure 1:**
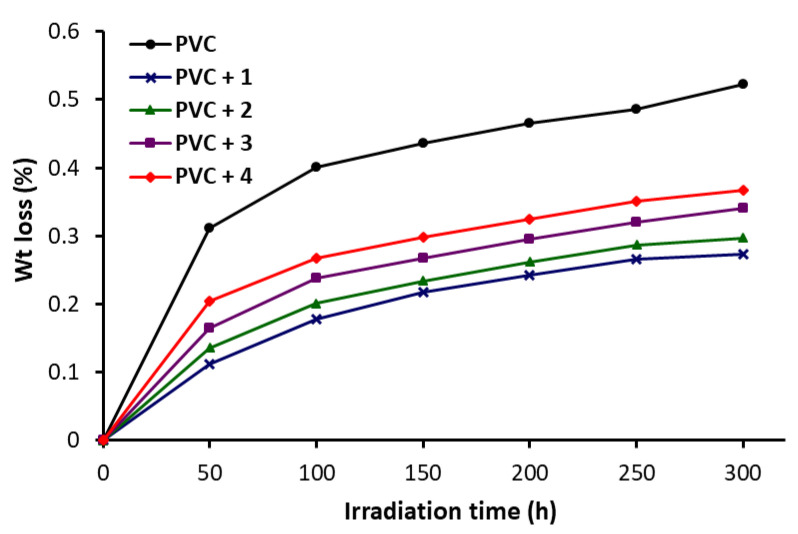
Effect of UV irradiation time on weight loss % of PVC films.

Photooxidation, thermal decomposition, and photodegradation of PVC leads to hydrogen chloride (HCL) release and formation of polymeric chains containing double bonds (polyenes). The UV light absorption by PVC increases as more double bonds form (conjugated polyenes), leading to bond cleavage and formation of unsaturated fragments with a low molecular weight [44,45]. In addition, PVC develops discoloration, crosslinking, and scissions over time. The PVC mass loss becomes more noticeable as photodegradation proceeds. Therefore, the PVC photodegradation can be simply and efficiently assessed by measuring the mass loss. The PVC mass loss is the difference between the weight of non-irradiated (*W*_1_) and irritated (*W*_2_) film after a specific irradiation time. Complexes **1**–**4** were added to PVC at a concentration of 0.05% by weight and thin films (40 µm) were produced. Such a concentration has been proven to be optimal to provide the most PVC protection without altering the color [17,46]. The blank and blended PVC films were irradiated (50 to 300 h) at room temperature, and the weight loss (%) of materials due to photodegradation was measured for different irradiation times using Equation (1). The results obtained are plotted and presented in Figure 1.

*Weight loss* (*%*) = [(*W*_1_ − *W*_2_)/*W*_1_ × 100 (1)

Figure 1 shows that tin-naphthalene complexes **1**–**4** played a role in reducing the PVC weight loss due to UV irradiation. The weight loss (%) after 50 h of irradiation was 0.312 for the blank film compared with 0.112 to 0.204 in the presence of tin-naphthalene complexes **1**–**4**. After 300 h of irradiation, the weight loss (%) for the blank PVC film was higher (0.523) than those obtained in the presence of the complexes used (0.273–0.341). The lowest weight loss (%) was seen for PVC + **1** (0.273) followed by PVC + **2** (0.297), PVC + **3** (0.341), and PVC + **4** (0.367). It was clear that complex **1** was the most efficient PVC stabilizer due to its high degree of aromaticity (three phenyl groups). Aromatic rings and heteroatoms (sulfur, nitrogen, and oxygen) stabilized PVC through direct absorption of light or coordination with polymeric chains.

### 2.5. IR Spectroscopy of PVC

**Figure 2:**
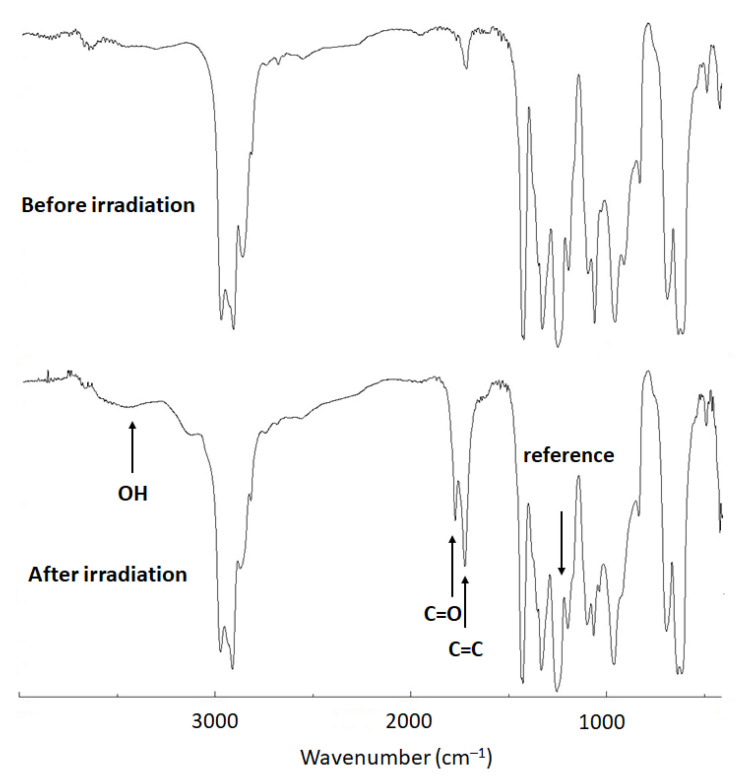
IR spectra of pure PVC film before and after irradiation.

**Figure 3:**
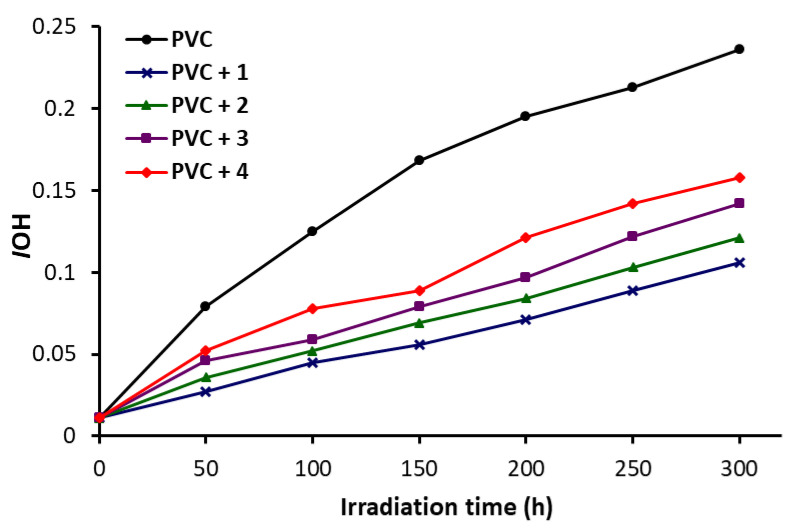
Effect of UV irradiation time on the *I*OH of PVC films.

**Figure 4:**
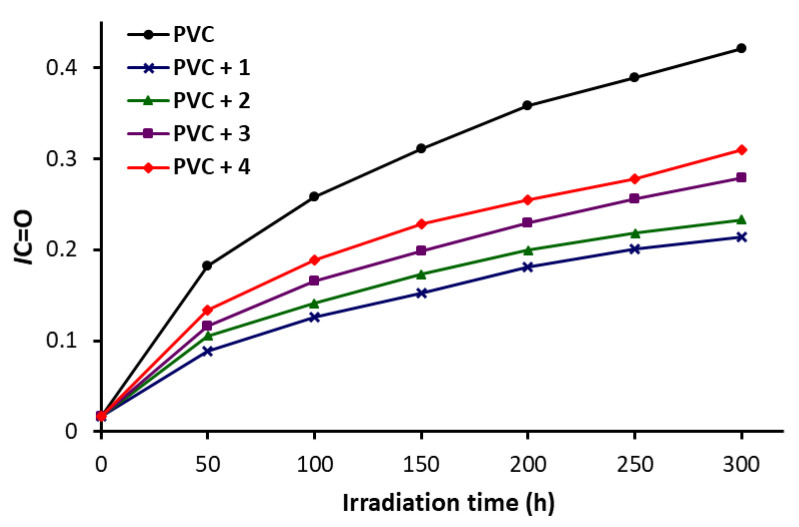
Effect of UV irradiation time on the *I*C=O of PVC films.

**Figure 5:**
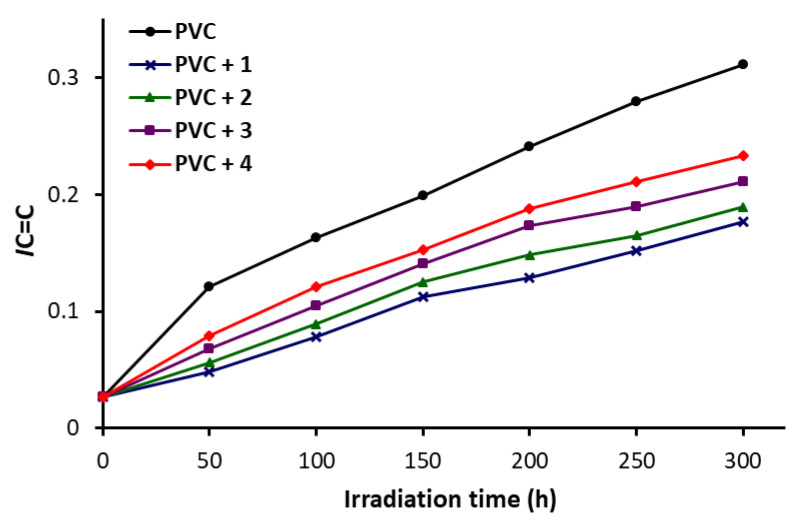
Effect of UV irradiation time on the *I*C=C of PVC films.

Irradiation of PVC with UV light leads to the formation of free radicals (e.g., Cl, –CH_2_–CH– radicals) [17,47]. These radicals are responsible for the formation of small polymeric fragments that contain double bonds (alkene, –CH=CH–). In the presence of oxygen, other fragments that contain carbonyl groups (e.g., ketone, –CH_2_–CO–) and hydroxyl residues (e.g., alcohol, –CH_2_–CHOH–) are produced. The use of IR spectroscopy can provide evidence about the growth in the intensity of peaks corresponding to the OH (3500 cm^−1^), C=O (1722 cm^−1^), and C=C (1602 cm^−1^) functional groups [48,49,50]. Therefore, the blank and blended PVC films were irradiated for different durations that ranged from 50 to 300 h. The IR spectra were recorded and the increase in the intensity of the peaks corresponding to hydroxyl, carbonyl, and alkene groups was measured. The IR spectra of pure PVC film before and after irradiation (300 h) are shown in Figure 2. The index for each functional group (*I_s_*) was calculated from the absorption of its peak (*A_s_*) and absorption of a reference peak (*A_r_*) using Equation (2) [51]. The reference peak corresponds to the –CH– bond (1328 cm^−1^) in PVC. The intensity of such a peak does not change during the irradiation process. The *I*OH, *I*C=O, and *I*C=C were calculated every 50 h, and the results obtained are shown in Figure 3, Figure 4 and Figure 5, respectively.

*I_s_ = A_s_*/*A_r_* (2)

Figure 3, Figure 4 and Figure 5 show a significant increase in the *I*OH, *I*C=O, and *I*C=C as irradiation time increased. The growth in functional group indices was very rapid in the first 50 h. The changes were smaller when PVC was blended with tin-naphthalene complexes compared with those obtained for the blank film. Again, the highly aromatic additive (i.e., complex **1**) was the most efficient PVC stabilizer. For example, the *I*C=C for the blank PVC was 0.331 compared with 0.177 for the film containing **1** at the end of the irradiation process. Such a result indicated clearly that the used complexes acted as efficient stabilizers for PVC and significantly reduced the elimination of HCl. Similarly, the *I*OH and *I*C=CO for the blank PVC was 0.236 and 0.421, respectively, compared with 0.106 and 0.214 for the blend containing **1**. The *I*OH and *I*C=CO patterns indicated that tin-naphthalene complexes are also able to reduce the photooxidation of PVC films upon irradiation.

### 2.6. Surface Morphology of PVC

**Figure 6:**
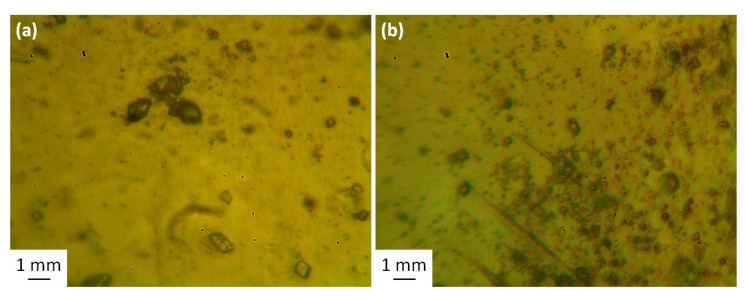
Optical microscopic images of PVC (blank) film: (**a**) before irradiation and (**b**) after irradiation.

**Figure 7:**
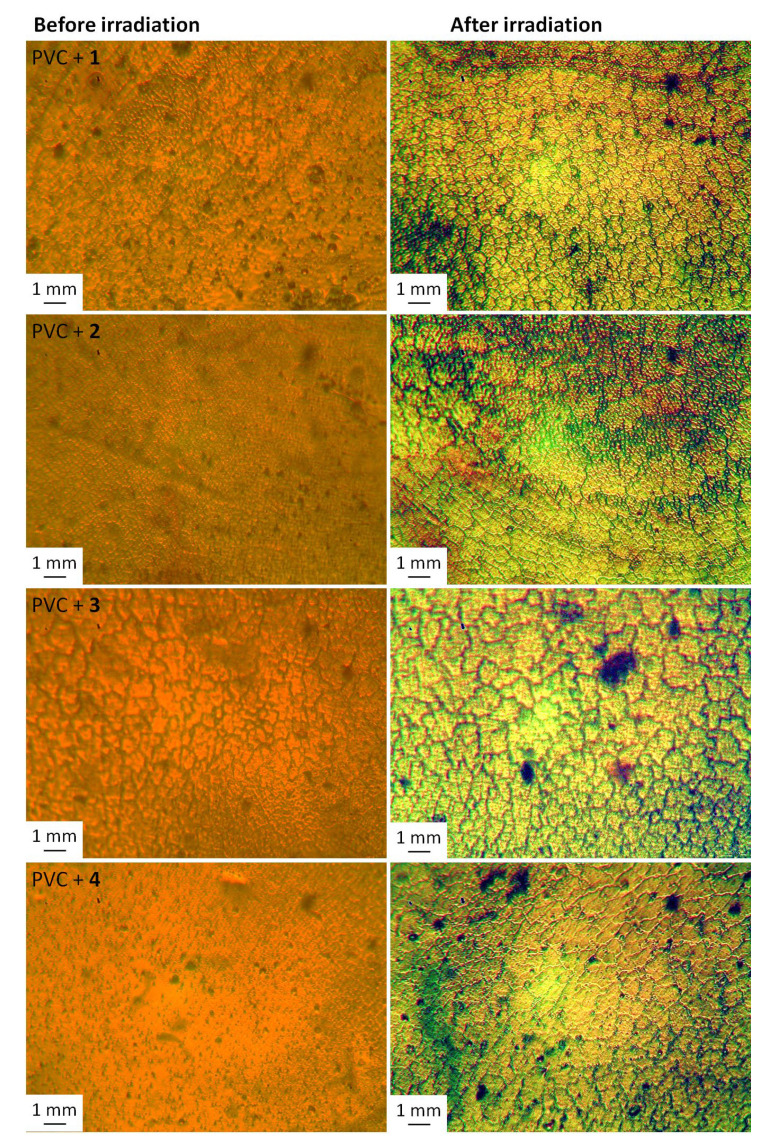
Optical microscopic images of PVC films before and after irradiation.

The role played by tin-naphthalene complexes **1**–**4** as stabilizers for PVC was investigated by examining the surface morphology of the irradiated films using optical microscopy. Optical microscopic images show any irregularities and roughness within the surface of PVC due to the release of HCl [52]. Figure 6 shows the optical images for the surface of irradiated and non-irradiated PVC (blank), and Figure 7 shows the optical images for the surface of PVC blended with tin-naphthalene complexes after irradiation. The irradiated PVC films showed rigid surfaces that contain a high number of cracks, white spots, and holes. For the PVC blended with tin-naphthalene complexes, the damage within the surface was less noticeable compared with that of the blank film. The tin-naphthalene complexes have the ability to act as scavengers for HCl and therefore reduce its negative effect on the PVC surface.

**Figure 8:**
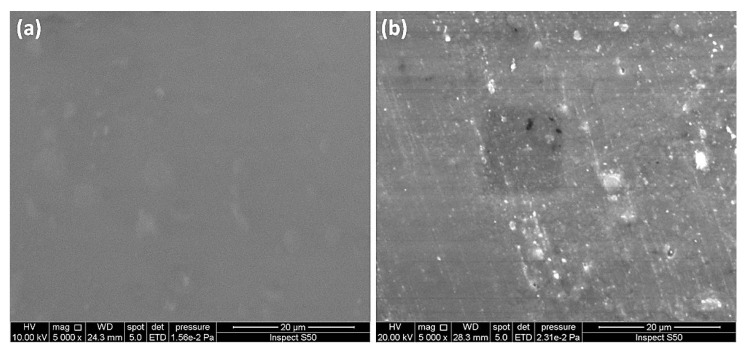
SEM images of the blank PVC film: (**a**) before irradiation and (**b**) after irradiation.

**Figure 9:**
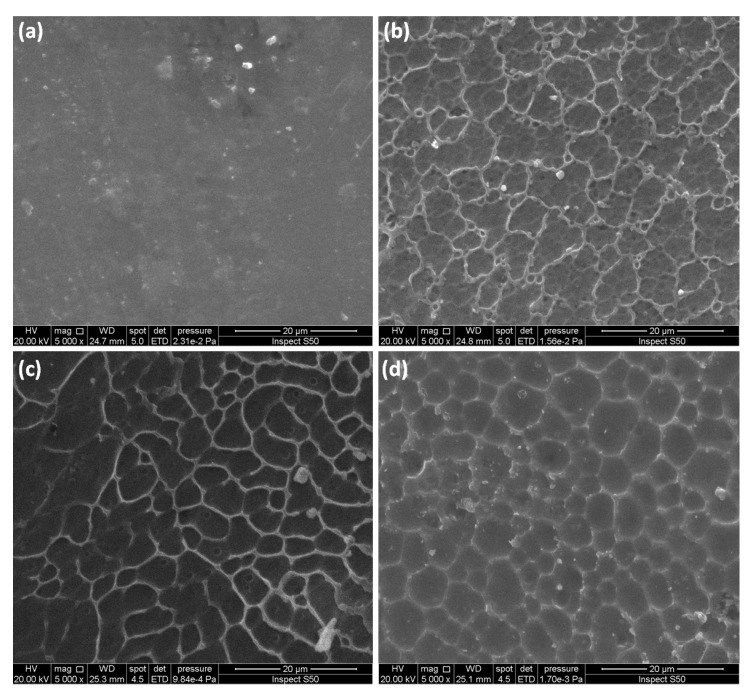
SEM images of PVC films after irradiation: (**a**) PVC + **1**, (**b**) PVC + **2**, (**c**) PVC + **3**, and (**d**) PVC + **4**.

**Figure 10:**
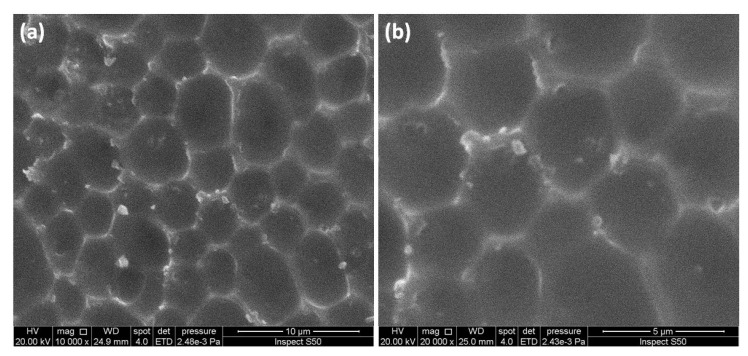
SEM images of PVC + **4** at: (**a**) 10 µm and (**b**) 5 µm.

Scanning electron microscopy (SEM) was used to investigate the variations that occurred within the surface of irradiated PVC [53]. The SEM images for the blank PVC films before and after irradiation are shown in Figure 8, whereas those blended with complexes **1**–**4** are shown in Figure 9. The surface of the PVC films was badly damaged in the case in which no additives were used. However, the surface defects were minimal within the surface of the blended PVC films, which reflects the role played by tin complexes in reducing photodegradation. The defects that appeared within the surface of the films are mainly a result of the elimination of volatiles (e.g., HCl) and the cross-linking of chains. The surface of the PVC + **1** (Figure 9) was more or less smooth and regular, with a small number of cavities. The SEM images of the PVC films containing complexes **2**–**4** showed a regular particle aggregation. The pores within the surface were hexagonal and appeared as honeycomb like-structures, particularly in the presence of complex **4** (Figure 10). A similar phenomenon was observed for the irradiated PVC blended with a dithiazole Schiff base in the presence of nickel chloride [20]. The formation of the honeycomb structure could be due the slow dehydrochlorination process and the coordination of the additives with the polymeric chains [54]. By comparison, the irradiated PVC containing a melamine Schiff base showed an ice-rock structure [35].

**Figure 11:**
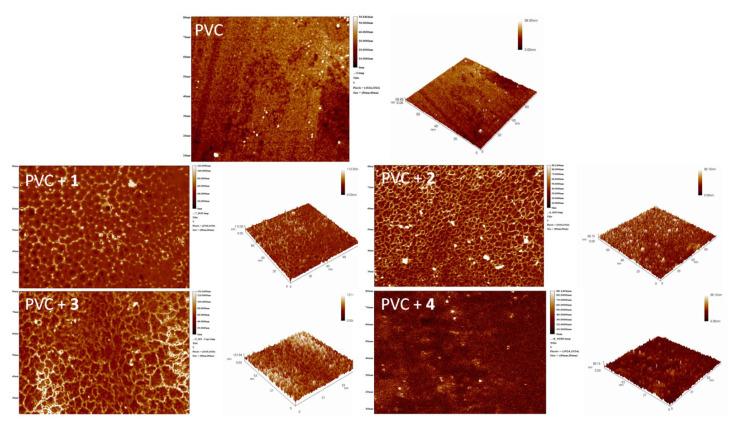
AFM images of irradiated PVC films.

The surface of the irradiated PVC blends was also investigated using atomic force microscopy (AFM). The AFM images show the smoothness and homogeneity of the surface without the need for a vacuum or electron beams [55]. Normally non-irradiated materials showed a surface with a high smoothness and homogeneity when inspected with AFM [56,57]. The AFM images (two- and three-dimensional) of the irradiated PVC films (300 h) are shown in Figure 11. These images indicate that the surface of the PVC blended with tin-naphthalene sulfonic acid complexes has a smoother and regular surface in comparison to the PVC (blank) film.

The roughness factor (*R*q) was very high (220) for the irradiated blank PVC film. The PVC films blended with tin complexes have a much lower *R*q compared with the blank one after irradiation. The *R*q was 3.7 for PVC + **1**, 10.3 for PVC + **2**, 11.4 for PVC + **3**, and 12.6 PVC + **4** films. Such a low *R*q for the blended films indicated that tin complexes significantly reduced the bond breaking [58] and dehydrochlorination [59] of PVC. The use of complex **1** led to a reduction in the *R*q by 59.5-fold, which is the highest ever reported. For example, the fold-reduction in *R*q of the irradiated PVC films was 5.2 over tin-naproxen complexes [60], 6.2 over tin-2-(4-isobutylphenyl)propanoate complexes [61], 6.6 over tin-furosemide complexes [62], 9.4 over tin-telmisartan complexes [63], and 16.6 over tin-ciprofloxacin complexes [64]. The use of Schiff bases led to a reasonable reduction (3.3–6.0) in the *R*q [65,66,67], whereas polyphosphate containing benzidine caused a reduction in *R*q by 16.7-fold [68]. The reductions obtained in *R*q (by fold) of irradiated PVC in the presence of various photostabilizers are summarized in Table 4.

### 2.7. Rate Constant of PVC Photodegradation

The efficiency of tin-naphthalene complexes **1**–**4** as PVC photostabilizers was tested further through the determination of the rate constant of photodegradation (*K*d).

As shown in Table 5, the *K*d value was highest (9.80 × 10^−3^ s^−1^) for the blank PVC film and lowest for the blend containing complex **1** (3.16 × 10^−3^ s^−1^). Clearly, the rate of photodegradation for the blank PVC was three times higher compared with the film containing **1**. All other complexes showed a significant reduction in *K*d (7.50–4.28 × 10^−3^ s^−1^) compared with that for the blank film. These results are consistent with those obtained from infrared, weight loss, specific functional groups’ formation, and surface morphology.

### 2.8. Suggested Mechanisms for PVC Photostabilization

**Figure 12:**
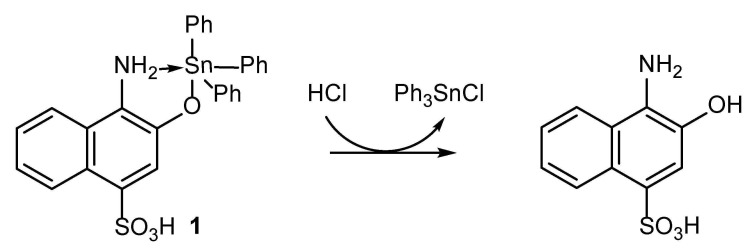
Complex **1** acts as a scavenger for HCl.

**Figure 13:**
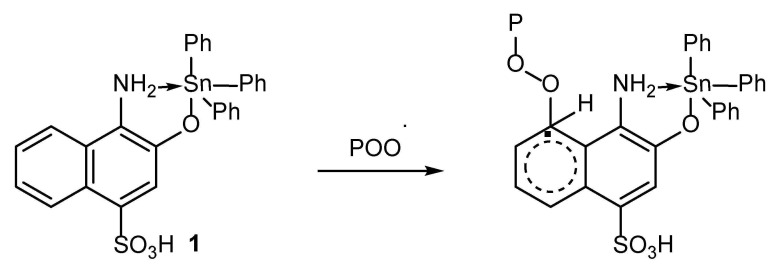
Complex **1** acts as a peroxide decomposer.

**Figure 14:**
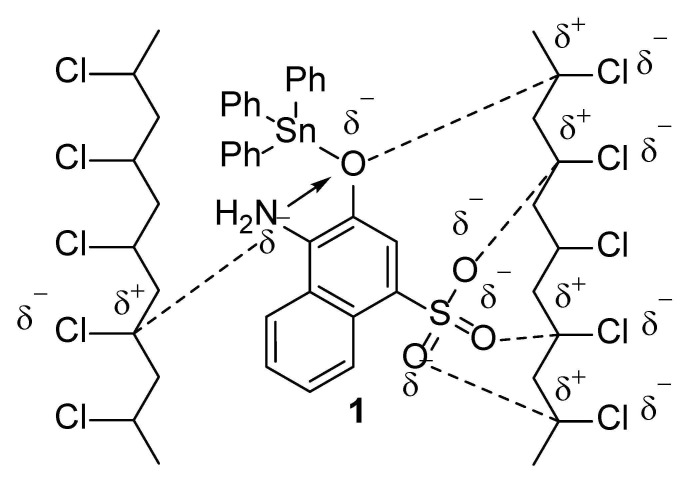
Attraction between PVC and Complex **1**.

Tin-naphthalene complexes **1**–**4** provided protection for PVC and reduced its photooxidation and photodegradation, in which **1** provided the greatest effect. Several mechanisms are proposed to highlight the role played by the complexes (particularly **1**) as stabilizers for PVC (Figure 12, Figure 13 and Figure 14). Complexes **1**–**4** contain an acidic center (tin atom) and therefore act as HCl scavengers (Figure 12) to produce substituted tin chloride along with the liberation of the ligand. The PVC photostabilization mechanisms are speculative based on scientific logic and previous reports [17,36]. However, it should be noted that the action of these additives within macromolecules or polymeric networks might be complex, and thus not simple or straightforward.

The additives used, and in particular complex **1,** act through scavenging peroxide radicals (Figure 13), which are produced due to degradation of PVC in the presence of oxygen. The excited state containing additives and peroxide radicals (chromophore) is highly stable due to resonance. Additionally, the coordination between additives and PVC can stabilize the polymeric chain (Figure 14).

## 3. Materials and Methods

### 3.1. Materials and Instrumentation

Chemicals and solvents were purchased from Merck (Schnelldorf, Germany). PVC with an average molecular weight of approximately 250,000 was purchased from Petkim Petrokimya (Istanbul, Turkey). A Vario EL III elemental analyzer (Elementar Americas Inc., Ronkonkoma, NY, USA) was used to determine the contents of carbon, hydrogen, nitrogen, and sulfur in the complexes. An FTIR-4200 spectrometer (Jasco, Tokyo, Japan) was used for recording the infrared spectra using a potassium bromide disc. The ^1^ H and ^119^ Sn NMR spectra were recorded on a Bruker DRX500 spectrometer (Zürich, Switzerland) in dimethyl sulfoxide (deuterated) at 500 and 149 MHz, respectively. The weathering tests were performed at room temperature on a Q-Panel tester (Homestead, FL, USA) and the surface morphology was examined using Inspect S50 (FEI Company, Czech Republic), KYKY-EM3200 (Ontario, CA, USA), and Meiji Techno (Tokyo, Japan) microscopes.

### 3.2. Synthesis of Trisubstituted Tin-Naphthalene Sulfonic acid Complexes ***1*** and ***2***

A stirred mixture of 4-amino-3-hydroxynaphthalene-1-sulfonic acid (1.20 g, 5 mmol) and triphenyltin chloride (1.93 g, 5 mmol) or tributyltin chloride (1.63 g, 5 mmol) in MeOH (60 mL) was refluxed for 4 h. The mixture was left at room temperature to cool down, and the solid produced was filtered, washed with MeOH, and dried to give complex **1** or **2** (Scheme 1) with a 75% or 70% yield respectively (Table 1).

### 3.3. Synthesis of Disubstituted Tin-Naphthalene Sulfonic acid Complexes ***3*** and ***4***

A stirred mixture of 4-amino-3-hydroxynaphthalene-1-sulfonic acid (1.43 g, 6 mmol) and dibutyltin dichloride (091 g, 2.5 mmol) or dimethyltin dichloride (0.66 g, 3 mmol) in MeOH (40 mL) was refluxed for 4 h. The mixture was left at room temperature to cool down and the solid produced was filtered, washed with MeOH, and dried to produce complex **3** or **4** (Scheme 2) with a 95% or 89% yield respectively (Table 1).

### 3.4. Preparation of PVC Films

The PVC films were prepared using the casting solution technique. To a stirred solution of PVC (5 g) in tetrahydrofuran (THF; 100 mL), tin complex (25 mg) was added. The solution was stirred for three hours then poured into a glass plate that contained 15 holes with a thickness of approximately 40 µm. The produced films were left to dry for a day at room temperature followed by eight hours at a reduced pressure using a vacuum oven.

### 3.5. Exposure to UV Light

The PVC films were exposed to UV light (365 nm) with a light intensity of 6.2 × 10^−9^ ein dm^−3^ s^−1^ nm for a period of time that ranged from 50 to 300 h at room temperature.

## 4. Conclusions

Four new tin-naphthalene sulfonic acid complexes were synthesized in good yields and their structures were elucidated. The tin complexes were added to PVC as additives and thin films were made. The tin complexes acted as photostabilizers for PVC upon the exposure to ultraviolet light for a long period of time. Various tools such as infrared analysis, weight loss, specific functional groups’ formation, surface morphology, and rate constant of photodegradation were used to test the effectiveness of the additives as photostabilizers. The complexes significantly reduced photodegradation and photooxidation of PVC by acting as peroxide decomposers, hydrogen chloride scavengers, and energy absorbers. In addition, the roughness factor of the surface for the irradiated films containing tin complexes was very low. The highly aromatic complex, which contains three phenyl substituents, showed the most stabilizing effect through efficient absorption of light energy.

## Figures and Tables

**Table 1:** Physical properties of tin-naphthalene sulfonic acid complexes **1**–**4**.

Complex	Color	Yield (%)	MP (°C)	Elemental Analysis (%) Calculated (Found)			
				C	H	N	S
**1**	Pale pink	75	213–215	57.17 (56.95)	3.94 (4.02)	2.38 (2.26)	5.45 (5.61)
**2**	Pale purple	70	150–152	50.02 (50.16)	6.68 (6.61)	2.65 (3.72)	6.07 (6.27)
**3**	Dark gray	95	166–167	47.40 (47.62)	4.83 (4.87)	3.95 (4.77)	9.04 (9.11)
**4**	Pale purple	89	243–245	42.26 (42.37)	3.55 (4.72)	4.48 (4.68)	10.26 (10.33)

**Table 2:** IR spectral data of tin-naphthalene sulfonic acid complexes **1**–**4**.

Complex	NH_2_	C–N	C–O	Sn–C	Sn–O
**1**	3244	1608	1528	517	455
**2**	3314	1611	1526	518	457
**3**	3240	1597	1530	515	468
**4**	3246	1611	1528	515	472

**Table 3:** ^1^ H and ^119^ Sn NMR spectral data (DMSO-*d_6_*; ppm, Hz) of tin-naphthalene sulfonic acid complexes **1**–**4**.

Complex	^1^ H	^119^ Sn
**1**	11.04 (1H, exch., s, SO_3_H), 9.78 (2H, exch., br s, NH_2_), 8.81 (1H, d, *J* = 8.1 Hz, Ar), 7.92 (1H, d, *J* = 8.1 Hz, Ar), 7.88 (1H, s, Ar), 7.58–7.37 (17H, m, Ar)	−174.0
**2**	11.06 (1H, s, SO_3_H), 9.84 (2H, exch., br s, NH_2_), 8.80 (1H, d, *J* = 8.0 Hz, Ar), 7.89–7.84 (2H, m, Ar), 7.59 (1H, app. t, *J* = 8.0 Hz, Ar), 7.42 (1H, app. t, *J* = 8.0 Hz, Ar), 1.62–1.59 (6H, t, *J* = 7.6 Hz, 3 CH_2_) 1.34–1.29 (6H, m, 3 CH_2_), 1.14–1.1 (6H, m, 3 CH_2_), 0.88 (9H, t, *J* = 7.6 Hz, 3 Me)	−167.0
**3**	11.01 (2H, s, 2 SO_3_H), 9.80 (4H, exch., br s, 2 NH_2_), 8.77 (2H, d, *J* = 8.2 Hz, Ar), 7.83–7.65 (4H, m, Ar), 7.42 (2H, app. t, *J* = 8.2 Hz, Ar), 7.28 (2H, app. t, *J* = 8.2 Hz, Ar), 1.62–1.54 (4H, t, *J* = 7.5 Hz, 2 CH_2_) 1.46–1.23 (8H, m, 4 CH_2_), 0.82 (6H, t, *J* = 7.5 Hz, 2 Me)	−274.1
**4**	11.03 (2H, s, 2 SO_3_H), 9.74 (4H, exch., br s, 2 NH_2_), 8.81 (2H, d, *J* = 8.0 Hz, Ar), 7.90–7.85 (4H, m, Ar), 7.58 (2H, app. t, *J* = 8.0 Hz, Ar), 7.41 (2H, app. t, *J* = 8.0 Hz, Ar), 0.69 (6H, s, 2 Me)	−242.5

**Table 4:** The effect of various additives on the *R*q of PVC films.

PVC Additive	R*q* Reduction in Fold	Reference
Tin-naphthalene sulfonic acid complexes	59.5	Current work
Tin-naproxen complexes	5.2	[57]
Tin-2-(4-isobutylphenyl)propanoate complexes	6.2	[58]
Tin-furosemide complexes	6.6	[59]
Tin-telmisartan complexes	9.4	[60]
Tin-ciprofloxacin complexes	16.6	[61]
Schiff bases of 1,2,3,4-triazole-3-thiol	3.3	[62]
Schiff bases of biphenyl-3,3′,4,4′-tetraamine	3.6	[63]
Schiff bases of melamine	6.0	[64]
Polyphosphate containing benzidine	16.7	[65]

**Table 5:** Rate constant of photodegradation (*K*d) of PVC films.

PVC Film	Rate Constant of PVC Photodegradation (*K*d; s^−1^)
PVC (blank)	9.80 × 10**^−3^**
PVC + **1**	3.16 × 10**^−3^**
PVC + **2**	4.28 × 10**^−3^**
PVC + **3**	6.30 × 10**^−3^**
PVC + **4**	7.50 × 10**^−3^**

## Data Availability

Data are contained within the article.

## Sample Availability

Samples of tin-naphthalene complexes are available from the authors.
